# Configurable web-services for biomedical document annotation

**DOI:** 10.1186/s13321-018-0317-4

**Published:** 2018-12-21

**Authors:** Sérgio Matos

**Affiliations:** DETI/IEETA, University of Aveiro, Campus Universitário de Santiago, Aveiro, Portugal

**Keywords:** Named entity recognition, Biomedical text mining, Web-services

## Abstract

The need to efficiently find and extract information from the continuously growing biomedical literature has led to the development of various annotation tools aimed at identifying mentions of entities and relations. Many of these tools have been integrated in user-friendly applications facilitating their use by non-expert text miners and database curators. In this paper we describe the latest version of Neji, a web-services ready text processing and annotation framework. The modular and flexible architecture facilitates adaptation to different annotation requirements, while the built-in web services allow its integration in external tools and text mining pipelines. The evaluation of the web annotation server on the technical interoperability and performance of annotation servers track of BioCreative V.5 further illustrates the flexibility and applicability of this framework.

## Introduction

The large amount of information and knowledge continuously produced in the biomedical domain is reflected on the number of published journal articles. In 2017, the PubMed/MEDLINE bibliographic database contained over 26 million references to journal articles in life sciences, of which more than one million were added in that year [1]. At this rate, staying updated with the current knowledge and identifying the most relevant publications and information on a given subject is a very challenging task for researchers. Similarly, it became unfeasible for curators of domain databases to manually find, extract, validate and structure relevant information published in the literature [2, 3]. To accelerate the curation process, automatic information extraction tools have been developed and integrated in the curation pipeline [4]. These tools apply information retrieval and ranking methods to expedite the identification of relevant literature, given particular curation requisites, and information extraction methods that identify textual mentions of entities (e.g. names of genes) or relations (e.g. interactions between a protein and a chemical). This then led to the creation of end-user interfaces to facilitate the use of these tools and to provide straightforward and efficient ways of validating, correcting or completing the automatic annotations. The success of the BioCreative Interactive Annotation Task series demonstrates the importance of these efforts [5].

The BioCreative [6, 7] community has promoted several shared tasks focused on document classification and triage, entity recognition (e.g. genes, chemicals) and relation extraction (e.g. protein-protein interactions, chemical-disease associations), which have contributed to the development and evaluation of biomedical information retrieval and extraction tools. Following those important achievements, the technical interoperability and performance of annotation servers (TIPS) task, part of BioCreative V.5, evaluated the technical aspects of inter-operable web services for entity recognition and document annotation [8].

In this paper we present the latest developments of Neji, an open-source modular framework for biomedical text processing and concept recognition, namely the in-built support for REST web-services. Additionally, Neji was added with capabilities for digital text extraction and annotation of PDF documents, which are also accessible through the web services. Neji web server was evaluated through participation in the TIPS task with a concept recognition service configured for annotating eight concept types through five dictionaries and three machine-learning models.

## Methods

The web services platform was built over Neji, providing a RESTful API that facilitates the use of the framework’s document annotation functionalities and an easy and intuitive web interface to define and manage annotation services. The distributed software package includes an embedded web server, easing its deployment.

### Neji

Neji is an open source framework for biomedical concept recognition built around four crucial characteristics: modularity, scalability, speed and usability [9]. Neji can be used as a software library or as a tool through its command line interface (CLI). It integrates several state-of-the-art methods for biomedical natural language processing (NLP), namely methods for sentence splitting, tokenization, lemmatization, POS, chunking and dependency parsing. The concept recognition tasks are performed using dictionary matching or machine learning techniques with normalization through dictionaries. The machine learning component makes use of MALLET [10] for training and applying conditional random fields (CRF) models [11], and provides simple regular-expression based methods for feature extraction which can be easily modified or extended. Dictionary-matching is based on efficient regular expression matching with Deterministic Finite Automatons (DFAs), using the implementation in [12]. Neji dictionaries are tab-separated files with two fields, as illustrated by the example in Listing 1: concept identifier, following the format “source:identifier:type:group”, and the list of synonyms for that concept, concatenated with a pipe (“|”). The semantic types and groups may follow a reference taxonomy such as the UMLS Metathesaurus or be user-defined. This simple format facilitates the creation of custom dictionaries, which can be compiled from any domain vocabulary, ontology or other lexical resource according to the user needs. The composed identifier facilitates grouping or filtering the matched terms at various levels: by concept identifier, by semantic type or by semantic group.

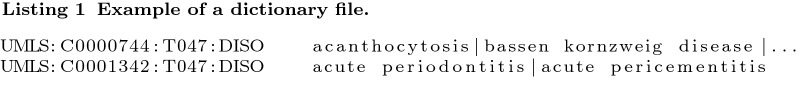



The architecture of Neji allows users to configure the processing of documents according to their specific objectives and goals, for example by simply combining existing or new modules for reading, processing and writing data, or by selecting the appropriate dictionaries or machine learning models according to the concept types of interest. Input (Reader) and output (Writer) modules offer off-the-shelf support for several formats including the most popular ones in biomedical text mining, such as IeXML, Pubmed XML, A1, CONLL and BioC, and facilitate extension to other formats.

The latest version of the framework includes various additions and improvements, namely:

- Neji web server—allows easy creation and management of several annotation services and provides a REST API for each service
- Machine learning module—now integrates Gimli [13] for training CRF models, eliminating the need to use a separate tool
- New input and output formats, including BioC and PDF files
- Improvements in performance, stability, and SDK usability

### Neji web services

The Neji web services platform facilitates the use and access to Neji functionalities by providing an easy and intuitive web solution to manage and use annotation services. The RESTful API allows developers and researchers to send their input documents and receive the annotation results. Besides Neji features, the web services platform offers also some other features:

- Management of concurrent annotation services. Allows an admin to create, edit and delete one or more annotation services;
- Flexible configuration of annotation services. Each service has its own resources (dictionaries and ML models) and properties;
- Pre-loading of resources: When a resource is added to the server, it is immediately loaded into the server memory. Therefore, on an annotation request it is not needed to wait for the load of any resource because they are already ready to be used, reducing the annotation time;
- Simple and intuitive user interface for management and annotation;
- Cross-platform and cross-browser support.

**Fig. 1:**
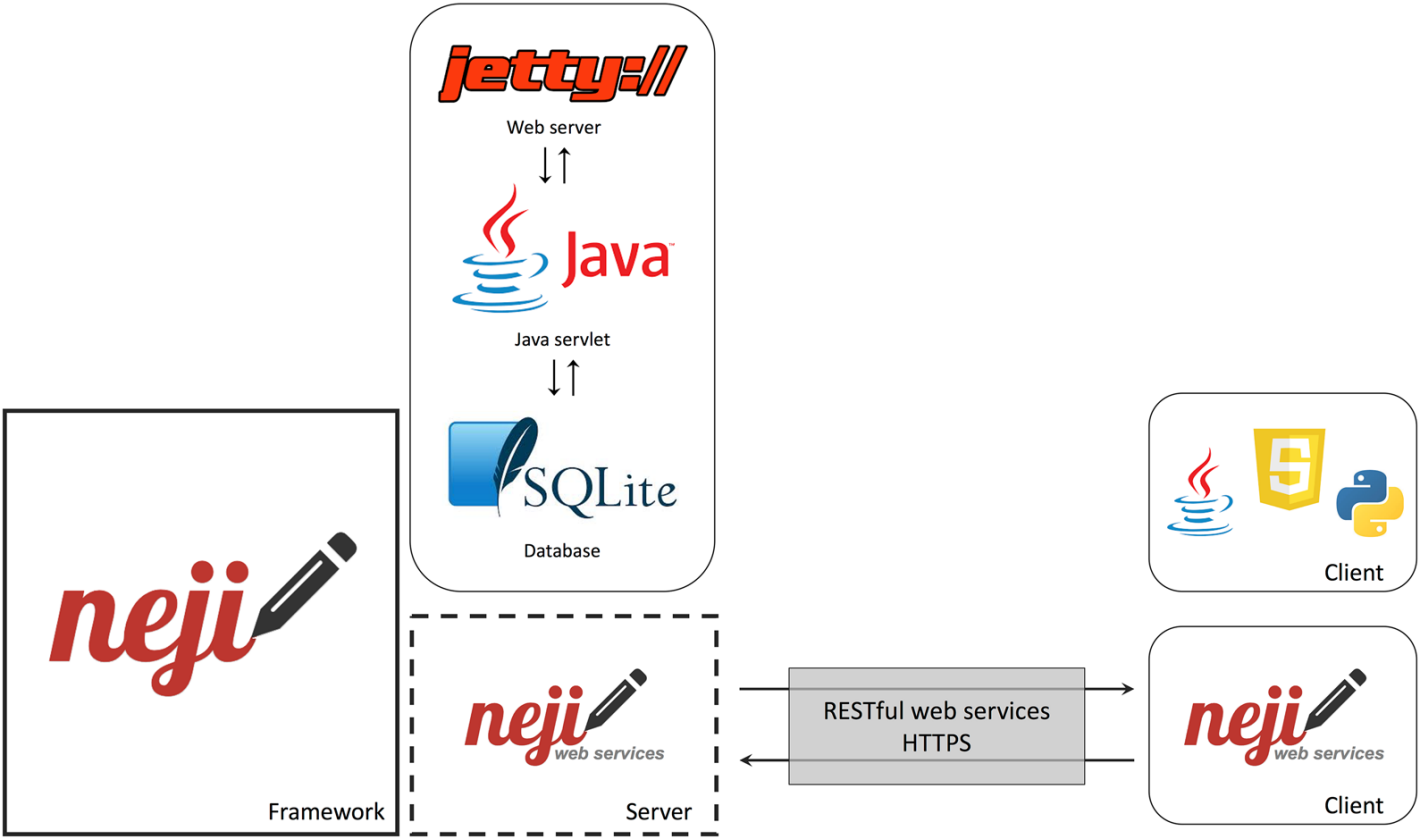
General architecture diagram. High-level view of the Neji web services architecture, built on top of the Neji framework and including an embedded web server and a light database for storing services and resource information. The Neji client offers management and document annotation user interfaces. Other client applications can access the document annotation functionalities through the REST web services

**Fig. 2:**
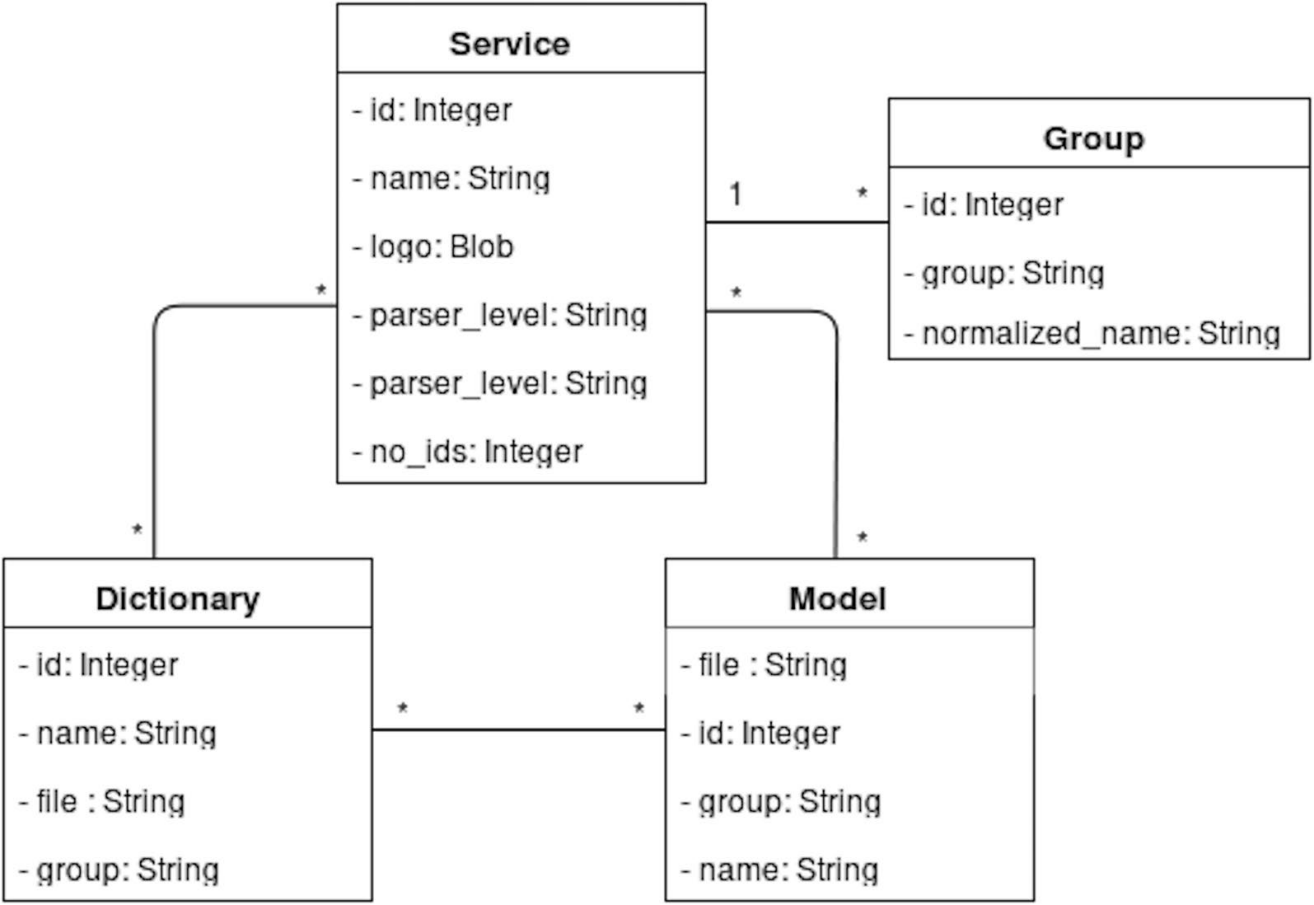
Neji web services data structure. The database stores information regarding the dictionaries and machine learning models available in the server and the web service configurations

Figure 1 illustrates the architecture of Neji web services platform. A light database is used to store the web service configurations and the dictionary and machine learning resources available in the server. Figure 2 shows the data structure that stores this information.

In the provided web interface, an administrator and a common user have different permissions. An administrator can add, edit and remove resources from the server, create and manage new annotation services, and annotate documents using the provided web services or annotation interface. A common user can not add resources or create new annotation services, but can use any of the provided annotation services, using both the web services and annotation interface.

#### Resources

**Fig. 3:**
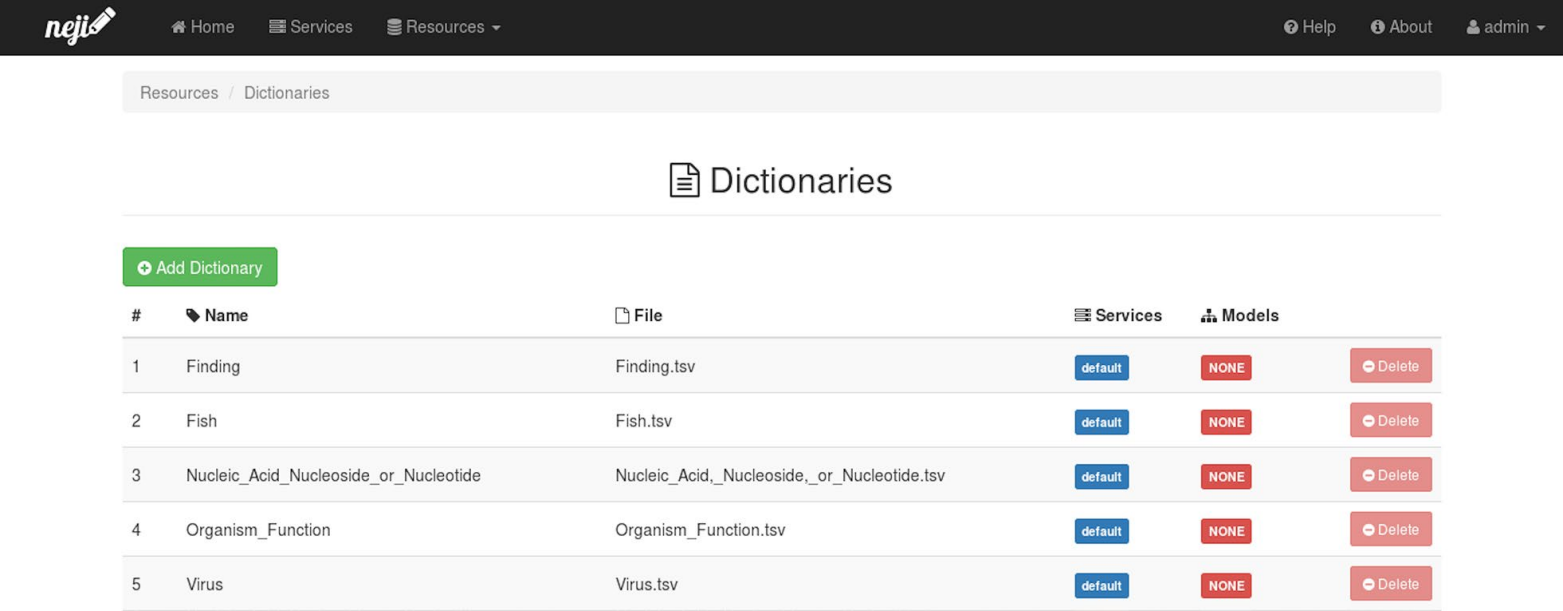
Neji web services dictionaries page. All dictionaries loaded in the server are listed in this page, identifying the annotation services that use them. New dictionaries can be added by simply uploading a new dictionary file

In the dictionaries page (Fig. 3) administrators can see a list of all dictionaries loaded in the server. For each dictionary the following information is provided: name, original file name, list of services that use it in the annotation process and list of models that use it in the normalization process. New dictionaries can be added and deleted in this page. When a new dictionary is added, the dictionary file is uploaded to the server and assigned with the name provided by the user.

**Fig. 4:**
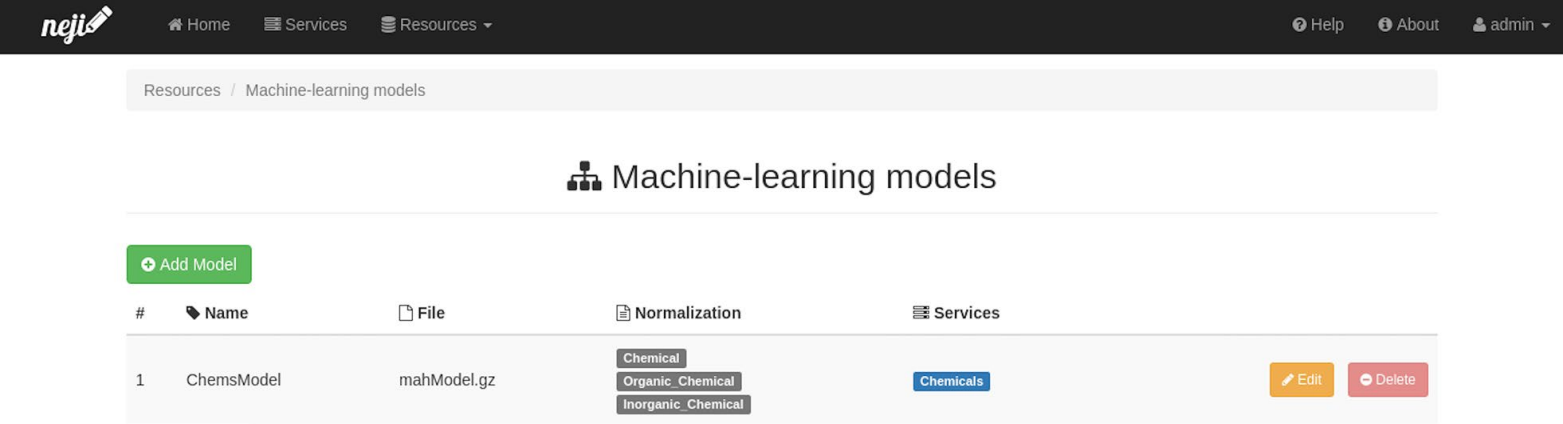
Neji web services models page. All models loaded in the server are listed in this page, identifying the annotation services that use them. New models can be trained with Neji and added to the server by simply uploading the model files

Similarly, in the machine-learning (ML) models page (Fig. 4) administrators can see a list of all loaded ML models in the server. For each model the following information is provided: name, original file name, list of normalization dictionaries and list of services that use it in the annotation process. New models can be trained with Neji, through the programming API or command line interface [9] and added to the server. When a new model is added, the model files are uploaded to the server and associated with the selected normalization dictionaries.

#### Services

**Fig. 5:**
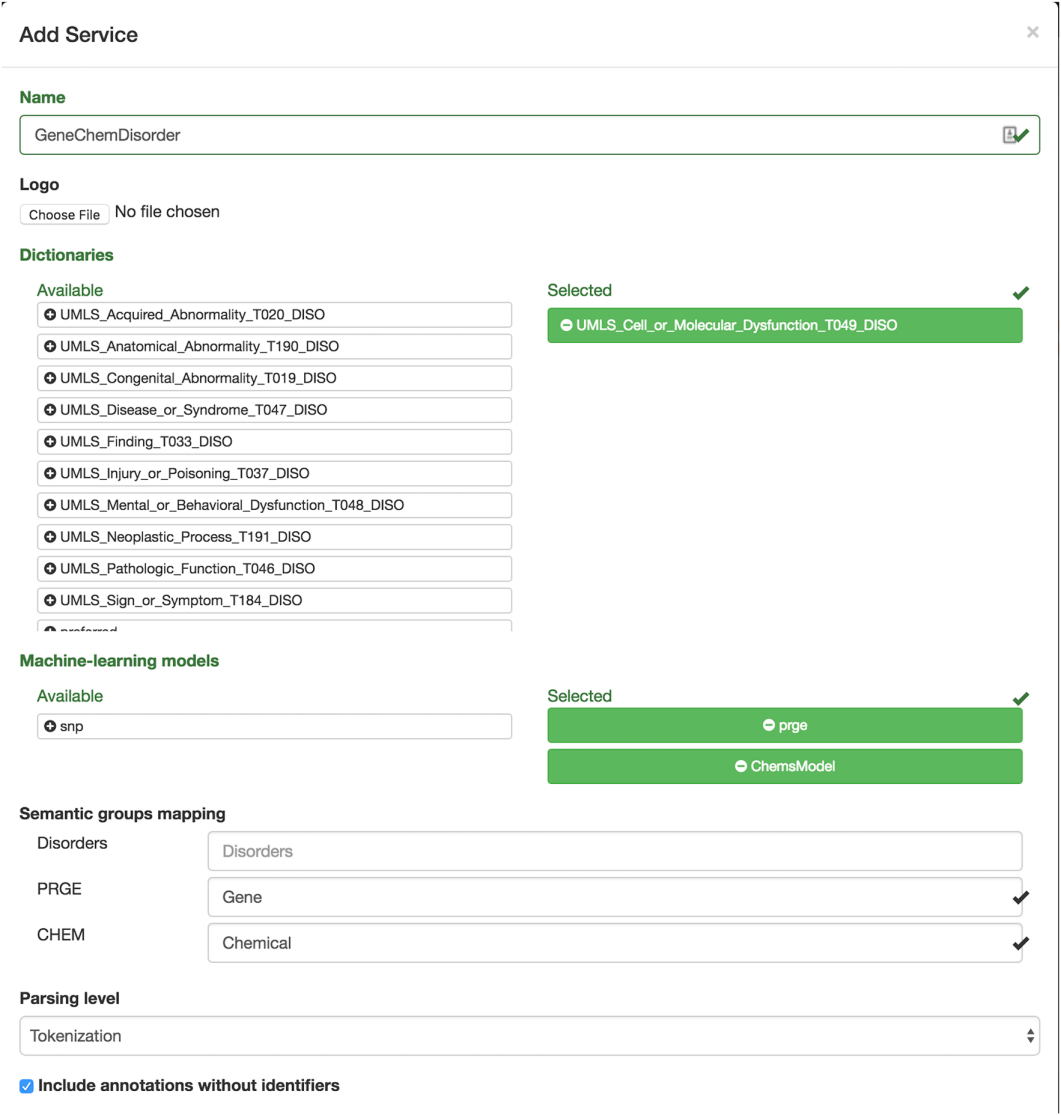
Neji web services new service form. A simple form is provided to define new annotation services using the dictionary and ML models previously added to the server

The services page allows administrators to see and edit all active services running in the server. To add a new annotation service an administrator needs to select the dictionaries and models to be used in the annotation process, selected from the list of resources available in the server, and define the level of linguistic parsing (from tokenization to dependency parsing) according to the type of features used by the ML models selected (Fig. 5). Additionally, the service name, an image or logo to identify the service, and if the annotation result should include the annotations that could not be normalized to an identifier, should be defined. Since the dictionary and ML normalization add a semantic group identifier to the annotations, an optional mapping is provided to map the resulting group identifiers to the desired name. One a new service is set up, it it automatically started and available for use.

#### Annotation

**Fig. 6:**
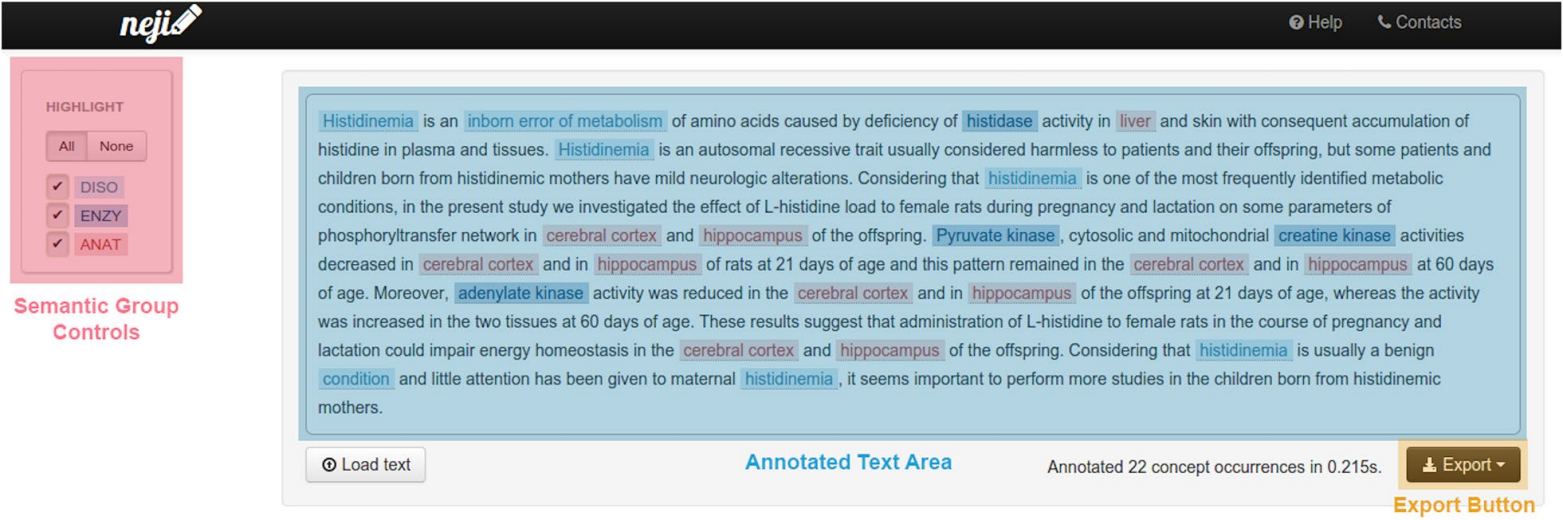
Neji web services annotation page. This page allows visualizing annotations and testing the created web services. Non-expert users can use this page to annotate small numbers of documents, exporting the results to a number of possible output formats

The annotation service page can be accessed by anyone. These are accessed through a general hyperlink, composed by the website domain and the service name. For example, if the name of the service is ‘Chemicals’, then the hyperlink for that page is https://neji-web-services-domain.com/annotate/Chemicals.The annotation page, based on the interface of Becas annotation tool [14], is presented in Fig. 6 and contains two major areas:

- Semantic groups control: allows the selection of the entity groups that should be recognized and annotated. One semantic group needs to be selected in order to perform the annotation. Once the annotation is performed, these buttons toggle the highlighting of each semantic group;
- Text box and input/output controls: allows selecting a PubMed article identifier, uploading a file, or pasting text to annotate, and displays the annotation result. The annotation results can be exported to a number of formats.

#### Web services

The developed RESTful API offers a set of web services that allow an easy and fast annotation of plain texts and PDF documents.

##### Annotate text web service

The annotate text web service can be accessed through an endpoint like https://neji-web-domain.com/annotate/[servicename]/annotate/, where [service name] is the name of the service that should be used to annotate the text. Table 1 contains the service parameters. The response is a JSON object structured as shown in Table 2.

**Table 1:** Parameters of annotate text web service

Name	Type	Default	Description
text	string: “...”	None (required)	Text to annotate
groups	object: {“GROUP”:true—false, ...}	If omitted: all groups true; If any groups present: all others false	Types of concepts to annotate

**Table 2:** Response of annotate text web service

Name	Type	Description
text	string: “...”	Submitted text
entities	array of strings: [“...”, ...]	Concepts recognized in text
ids	object of objects: {“CID”: {“name”: “...”, “refs”: [“...”, ...], ...}	Concept metadata, with concept IDs

##### Annotate PDF document web service

The annotate PDF document web service can be accessed through an endpoint like https://neji-web-domain.com/annotate/pdf/annotate/[servicename]/, where [service name] is the name of the service that should be used to annotate the text of the PDF. Table 3 contains the service parameters. The result is the same as for the plain text annotation.

**Table 3:** Parameters of annotate PDF document web service

Name	Type	Default	Description
pdf_file	file	None (required)	PDF file to extract text
groups	object: {“GROUP”:true—false, ...}	If omitted: all groups true; If any groups present: all others false	Types of concepts to annotate

##### Export web service

Export web service can be accessed through a endpoint like https://neji-web-domain.com/annotate/[servicename]/export/, or https://neji-web-domain.com/annotate/pdf/[servicename]/export/ for PDF documents, where [service name] is the name of the service that should be used to annotate the text. The service parameters are the same as for the corresponding annotation service, plus an additional parameter *format* (type string) to identify the output format. The response are the annotation results in the selected output format.

##### Extract PDF text web service

An additional service is provided though an endpoint like https://neji-web-domain.com/annotate/pdf/extract/ to extract the full text of a PDF file, which is the only service parameter.

**Fig. 7:**
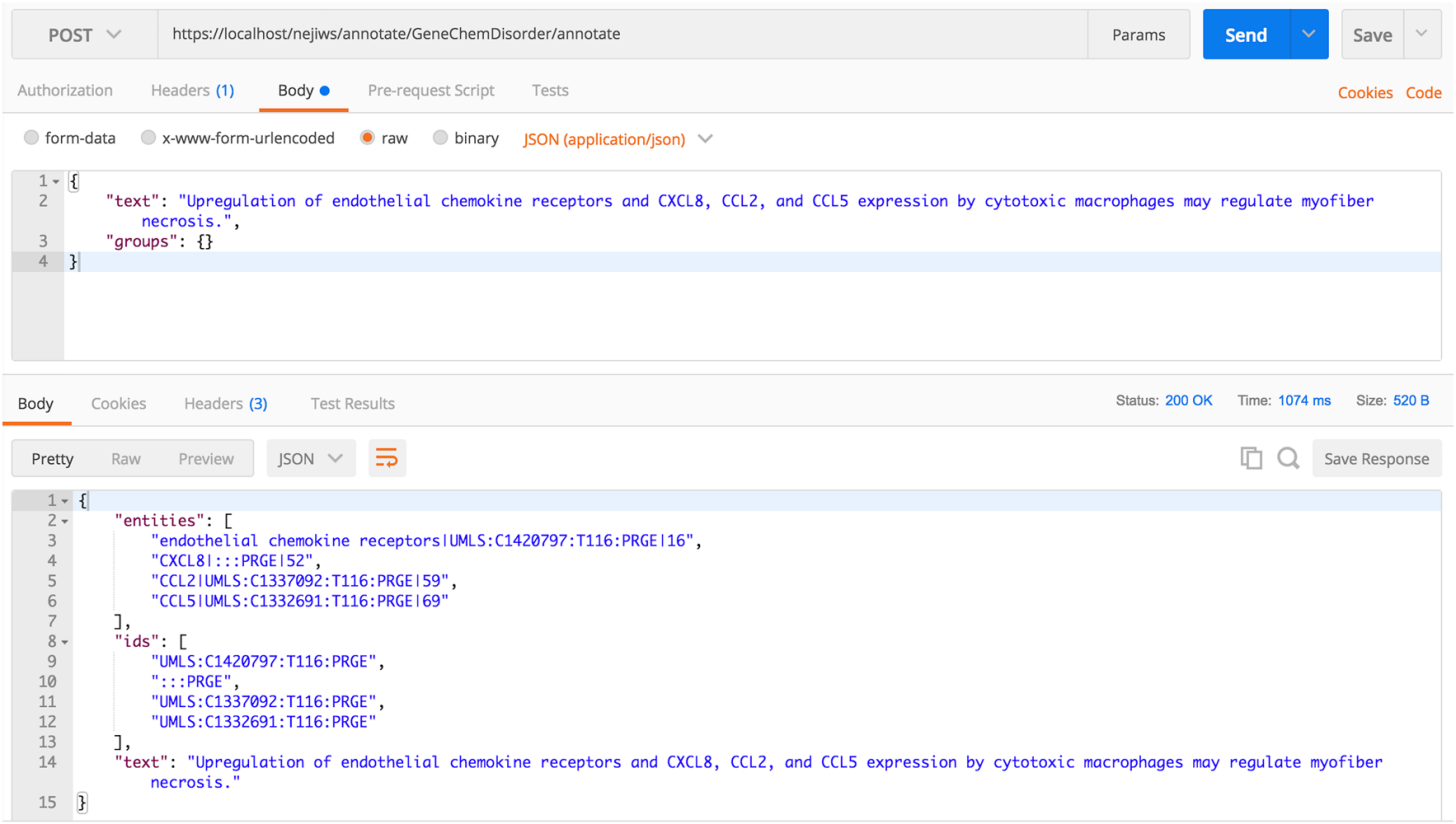
Example plain text annotation request. The figure illustrates an example of using a web service, with service name *umls*, to annotate a sentence in plain text. The *Chemicals* and *GenesAndProteins* semantic groups were selected

Figure 7 shows an example of using the plain text annotation web service.

These functionalities are also available through the programming API, provided by the developed Java library and Python module. Listing 2 presents an example of using the Java library to annotate a PDF document.

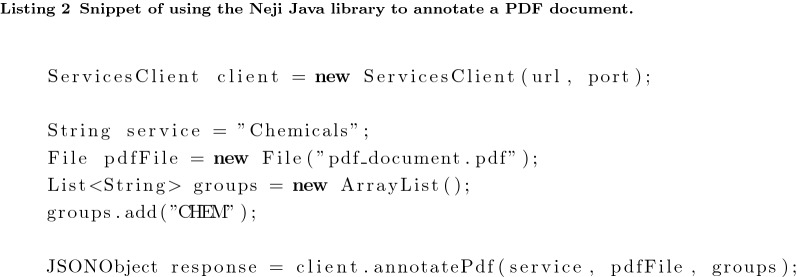



###### TIPS task

We evaluated the flexibility and applicability of Neji web services in the technical interoperability and performance of annotation servers task [8]. For this, we developed four new writer modules to support all the output formats proposed in the task, namely TSV, JSON, BioC and BioC JSON. Additionally, the REST API was extended and adapted according to the task requirements.

An annotation service was configured that allows annotating the following concept types: Anatomic Component, Diseases, Subcellular structure, Tissue and Organ, and Organism, through dictionaries compiled from the UMLS Metathesaurus, as described in [9], Chemicals and Genes/Proteins, through machine learning models trained on the BioCreative V CHEMDNER corpus [15], and Mutations, using an ML model trained on the tmVar corpus [16]. The server accepts raw text as input, as well as PubMed and PubMedCentral identifiers, which are used for obtaining the documents to be processed. The output format and annotated concept types can be configured by using the custom API parameters, as shown below. By default, all concept types are returned.

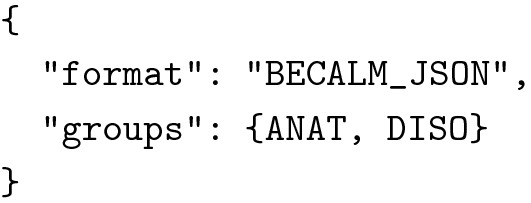



## Results and discussion

Neji has been evaluated on several corpora, covering different concept types [9, 17, 18]. Table 4 shows a summary of the concept identification performance.

**Table 4:** Neji concept recognition results on a variety of corpora and concept types

Corpus	Concept type	F-score (%)	Method
CRAFT [19]	Species [9]	95	D
	Cell [9]	92	D
	Gene and protein [9]	76	ML
	Chemicals [9]	65	D
	Cellular component [9]	83	D
	Biological process and molecular function [9]	63	D
NCBI Disease [20]	Disorders [9]	85	D
Anem [21]	Anatomy [9]	82	D
BC II gene mention [22]	Gene and protein [13]	87	ML
tmVar [16]	Genetic variants [18]	86	ML
BC IV ChemdNER [15]	Chemicals [17]	87	ML
BC V.5 CEMP [23, 24]	Chemicals [25]	87	ML

D dictionary; ML machine-learning

The annotation service for participating in the TIPS task was configured to run with 23 threads and was deployed on a Docker container with 32 GB of memory running on a server with 24 processing cores.

We performed a simple evaluation in terms of processing times by submitting several requests to the server, with different number of documents. We followed the procedure defined for the TIPS task [8], in which the document text is obtained from the BeCalm abstract and patent servers, and measured the time since the request was submitted to the Neji annotation service until the annotation results were returned. We observed average processing times ranging from 11.5 s for abstracts and 9.35 s for patents when annotating a single document, to 0.347 s per abstract and 0.173 s per patent when annotating sets of 1000 documents (Table 5).

**Table 5:** Average processing times, in seconds, for documents obtained from the BeCalm document servers

No. documents	Abstracts	Patents
1	11.5	9.35
100	0.421	0.236
1000	0.347	0.173

We also measured the processing time for documents sent directly to the annotation server, that is, without request to the BeCalm document servers. In these tests, the full Craft corpus [26], composed of 67 full text documents containing more than 560,000 tokens in total, was annotated in 15 minutes, which corresponds to an average processing time of 13.55 s per document and a processing speed over 600 tokens per second. Documents were sent to the annotation service one at a time and as raw text.

## Conclusions

Various biomedical information extraction tools have been proposed and made publicly available to the community, some of which are offered as open-source. Nevertheless, there are still difficulties when these tools need to be used by non-experts or integrated in text mining pipelines. Furthermore, while some web-services are available that allow annotating texts without the need for complex setups or computational resources [14, 27], the fact that these are centrally managed constitutes a limiting factor for some types of users.

This paper describes an open-source solution, part of the Neji framework for biomedical text processing and concept recognition, for easily configuring, deploying, and using text annotation services. Using the provided framework, expert and non-expert users can easily add their own dictionaries, following a simple tabular structure, and trained models, and configure web services that use any combination of these resources for annotating text, export the results to a number of formats or integrate the services in their annotation pipelines. Extensibility of the solution is provided by the modular architecture of Neji, that serves as processing backend. Additionally, Neji provides simple ways of training CRF machine learning models that can be directly used in the web annotation services.

Neji achieves high annotation accuracy for different semantic groups, as evaluated in several standard corpora and demonstrated in previous works [9, 17, 18]. The web services were evaluated through participation in the TIPS task, achieving annotation speeds of nearly 3 abstracts per second when annotation eight different concept types using five dictionaries and three machine-learning models.

## Abbreviations

CLI: command line interface
CRF: conditional random field
DFA: deterministic finite automaton
ML: machine-learning
NLP: natural language processing
TIPS: technical interoperability and performance of annotation servers
